# Beyond the pump: integrating the heart’s endocrine function into early medical education

**DOI:** 10.1080/10872981.2026.2704285

**Published:** 2026-07-15

**Authors:** JC Malsawmzuali, Fadi W. Adel, Wojciech Pawlina, Punnose Kattil

**Affiliations:** a Department of Cardiovascular Medicine, Mayo Clinic, Rochester, Minnesota, USA; b Department of Clinical Anatomy, Mayo Clinic, Rochester, Minnesota, USA

**Keywords:** Cardiac endocrine function, natriuretic peptides, medical education, curriculum integration, authentic learning

## Abstract

**Background:**

The heart is commonly introduced in early medical education through its structural, mechanical, and electrophysiological roles. Although this framing remains foundational, it may underemphasize the heart’s endocrine functions. Cardiac endocrine signaling, particularly natriuretic peptide physiology, offers a concise structure-function example linking anatomy, histology, cardiovascular physiology, and clinical biomarker interpretation.

**Objective:**

To synthesize scientific and educational principles of cardiac endocrine function and propose an anatomy-anchored framework for integration into pre-clerkship medical education.

**Methods:**

A librarian-assisted narrative review was conducted in May 2025 using representative literature from PubMed, Google Scholar, and Scopus, supplemented by historical, molecular, physiological, anatomical, and medical education sources. The search focused on cardiac endocrine function, natriuretic peptides, and medical education and yielded 23 abstracts. The aim was conceptual synthesis rather than systematic or exhaustive retrieval.

**Results:**

The discovery of atrial natriuretic peptide (ANP), B-type natriuretic peptide (BNP) and C-type natriuretic peptide (CNP) established the heart as an endocrine organ influencing renal, and cardiovascular homeostasis. The available medical education literature provides limited explicit guidance on how this topic should be introduced, and reinforced in early curricula. Integration of cardiac endocrine biology aligns with principles of cognitive integration, spiral curriculum design, and authentic learning. ANP and BNP/NT-proBNP are proposed as high-yield teaching examples: ANP links atrial structure and stretch to volume regulation, whereas BNP/NT-proBNP links ventricular wall stress to heart failure evaluation.

**Conclusions:**

Early integration of the heart’s endocrine function into medical curricula may fortify structure-function reasoning, enhance systems thinking, and support mechanistic clinical reasoning. Rather than adding a separate curricular block, cardiac endocrine signaling can be incorporated through anatomy and histology, then reinforced in cardiovascular physiology, renal physiology, biochemistry, pharmacology, and clinical reasoning. Aligning foundational instruction with modern cardiovascular science can improve learners’ conceptual frameworks and better prepare them for clinical decision making.

## Introduction

The heart is commonly introduced in early medical education through its mechanical, structural, and electrophysiological roles: chambers, valves, great vessels, conduction pathways, and coordinated contraction [1,2]. This framing remains foundational, particularly within anatomical sciences and cardiovascular physiology, but it can obscure another well-established dimension of cardiac biology: the heart’s function as an endocrine organ [3–7]. Among cardiac derived hormones, atrial natriuretic peptides (ANP) and B-type natriuretic peptides (BNP) provide especially useful examples for medical learners because they connect cardiac structure, myocardial stretch, renal sodium handling, extracellular volume regulation, and clinical biomarkers of heart failure [6–8].

Historically, medical understanding of the heart has evolved from symbolic and anatomical interpretations to circulatory, mechanical, and electrophysiological models [9–11]. Although this history remains important, the discovery of cardiac endocrine signalling added a further conceptual shift: the heart is not only a pump and conduction system, but also a signalling organ that communicates with the kidney, vasculature, and neuroendocrine systems [3–7,9–19].

A pivotal moment in molecular cardiology came in the 1980s with the discovery of atrial natriuretic peptide (ANP), followed by B-type natriuretic peptide (BNP) and C-type natriuretic peptide (CNP) [3,4,6,7]. The discovery of these peptides revealed that heart contributes to the regulation of blood pressure, renal function, extracellular volume, and neuroendocrine homoeostasis through endocrine signalling pathways. ANP and BNP are secreted primarily by cardiac myocytes in response to myocardial stretch, volume expansion, or pressure overload, whereas CNP is produced largely by endothelial tissues and functions predominantly through local paracrine and autocrine mechanisms [3,4,6–8].

These concepts are particularly well suited to anatomy-centred medical education because the endocrine function of the heart is inseparable from its structure [20]. Atrial and ventricular myocyte organisation, myocardial wall stress, chamber-specific responses to pressure and volume, and histologic evidence of peptide-producing cells provide concrete opportunities to connect anatomy and histology with physiology, biochemistry, and clinical reasoning [6,8,21]. Rather than presenting cardiac anatomy only as the architecture of flow and conduction, educators can use natriuretic peptide biology to show how cardiac structure also supports endocrine communication with the kidney, vasculature, and neuroendocrine systems [6,8,22].

Clinically, natriuretic peptides are central to contemporary cardiovascular care. BNP and NT-proBNP are widely used in diagnoses and management of heart failure, assessing risk in valvular disease, and guiding treatment algorithms [6,8,23]. Natriuretic peptide biology could potentially help learners understand therapeutic strategies such as neprilysin inhibition, including sacubitril/valsartan, an angiotensin receptor-neprilysin inhibitor used in heart failure therapy [8,23,24]. When introduced alongside early cardiovascular, renal, endocrine, or anatomical concepts, these peptides can help students interpret dyspnoea, congestion, ventricular wall stress, and volume overload as consequences of underlying structure-function relationships rather than isolated facts to memorise [8,22,23].

Despite the scientific and clinical relevance of cardiac endocrine function, published medical education literature provides limited explicit guidance on how this topic should be integrated into early curricula. The extent to which this topic is taught likely varies across institutions, and this review does not claim that cardiac endocrine function is absent from medical education. Instead, it identifies an educational opportunity: making the heart’s endocrine role more explicit within pre-clerkship anatomy, histology, physiology, biochemistry, and clinical reasoning instruction [8,25].

This narrative review asks: how can the heart’s endocrine function, particularly natriuretic peptide physiology, be meaningfully integrated into early medical education? We synthesise representative literature from cardiovascular, anatomical, physiological, biochemical sciences and medical education to outline core concepts, clarify the educational rationale, and propose an anatomy-anchored framework for incorporating cardiac endocrine signalling into pre-clerkship curricula.

## Methods

This article is a narrative review informed by structured librarian-assisted literature searches conducted in May 2025. A narrative review approach was selected because the purpose was to synthesise biomedical, clinical, anatomical sciences, and medical education literature in order to develop a practical curricular framework. The intent was not to perform exhaustive evidence retrieval, formal risk-of-bias assessment, or quantitative synthesis. This approach is consistent with published descriptions of narrative reviews as syntheses that can address broader conceptual questions, organise evidence thematically, and generate educational or clinical insight while maintaining transparency regarding literature search [26–29]. We also used the domains emphasised in the Scale for the Assessment of Narrative Review Articles, including review importance, clear aims, description of the literature search, referencing, and scientific reasoning, as a quality guide during revision [30].

Searches were conducted in PubMed (National Library of Medicine/National Centre for Biotechnology Information), Scopus (Elsevier; pre-1996-present), and Google Scholar (Google LLC) for articles between the years 1985 and 2025, with earlier landmark sources included when identified through reference lists. Search terms included combinations of ‘cardiac endocrine function’, ‘heart as an endocrine organ’, ‘endocrine function of the heart’, ‘natriuretic peptides’, ‘atrial natriuretic peptide’, ‘ANP’, ‘B-type natriuretic peptide’, ‘BNP’, ‘C-type natriuretic peptide’, ‘CNP’, ‘anatomy education’, ‘histology education’, ‘cardiovascular education’, ‘basic science integration’, ‘medical education’, and ‘pre-clerkship curriculum’. Reference lists of key landmark articles were also reviewed to identify additional relevant sources [26,28].

The search combined terms for cardiac or myocardial endocrine function and natriuretic peptides with terms for medical education, medical curriculum, pre-clerkship or preclinical education, students, learners, and curricula. This targeted search retrieved 23 abstracts, which were reviewed for direct or adjacent relevance to the review question.

Inclusion criteria consisted of peer-reviewed publications that:

1. Described physiology, molecular mechanisms, or clinical applications of natriuretic peptides.
2. Described anatomy, histology, physiology, biochemistry, renal physiology, or cardiovascular foundations relevant to pre-clerkship medical education.
3. Addressed curriculum integration, basic science teaching, or mechanisms of clinical reasoning, knowledge transfer, authentic learning, or systems-based instruction.
4. Discussed teaching strategies relevant to integrating cardiac endocrine signalling into anatomical sciences or other foundational science courses.

Excluded materials included non-peer-reviewed sources, conference abstracts without sufficient details, and articles unrelated to either cardiovascular physiology, natriuretic peptide biology, anatomical sciences education, or medical education. Articles were selected for conceptual relevance rather than through systematic or protocol-driven screening. Titles, abstracts, and full texts were reviewed by the author team for relevance to the review question. Because this was a narrative review rather than a systematic or scoping review, inter-rater reliability, formal quality appraisal was not conducted nor was PRISMA-based reporting adopted.

The synthesis was organised iteratively around the following themes:

1. Core scientific concepts related to the heart's endocrine function.
2. Clinical relevance of natriuretic peptide physiology.
3. Limited explicit guidance in published medical education literature on how this topic should be taught.
4. Anatomy-anchored framework for integrating cardiac endocrine function into pre-clerkship curricula.

This review was not designed to quantify curricular coverage across medical schools or to determine whether cardiac endocrine function is absent from individual curricula. Rather, it synthesises available literature to identify a curricular opportunity and propose practical strategies for integration.

## The heart as an endocrine organ: scientific foundations

The heart’s endocrine function is primarily mediated by a family of structurally related peptides: ANP, BNP, and CNP. For early medical learners, these peptides provide a useful structure-function sequence: cardiac or vascular cells sense stretch or local physiological signals, release peptide messengers, and communicate with the kidneys, vasculature, and neuroendocrine systems through receptor-mediated cyclic guanosine monophosphate (cGMP) signalling [4,6–8]. This makes cardiac endocrine function especially relevant to clinically oriented medical education, because it links myocardial structure, chamber stress, histology, renal physiology, and clinical biomarker interpretation.

### Atrial natriuretic peptide (ANP)

ANP is a 28-amino acid peptide synthesised and stored in atrial myocytes and released in response to atrial stretch, typically caused by increased blood volume reflecting its role as a volume-sensitive cardiac hormone [6,7,21]. ANP promotes natriuresis and diuresis by increasing glomerular filtration rate and reducing sodium reabsorption in the renal collecting ducts. This in turn causes vasodilation, leading to reduced systemic vascular resistance and blood pressure [6–8].

ANP also suppresses key neuroendocrine systems involved in volume and pressure regulation, including the renin-angiotensin-aldosterone system (RAAS) and vasopressin release. These combined effects reduce preload and afterload, thereby decreasing cardiac workload [31,32].

### B-type natriuretic peptide (BNP)

BNP, a slightly larger 32-amino acid peptide, is produced primarily by ventricular myocytes and released in response to ventricular wall stress, such as pressure or volume overload [6–8]. For learners, BNP provides a direct bridge from ventricular anatomy and mechanics to clinical interpretation: ventricular stretch becomes a measurable circulating biomarker.

Functionally, BNP closely mirrors the action of ANP, promoting natriuresis, diuresis, vasodilation, and inhibition of RAAS activity [6,7]. Clinically, BNP and an inactive *N*-terminal fragment (NT-proBNP) are widely used in the evaluation of heart failure because circulating concentrations generally increase in response to myocardial wall stress and correlate with diagnostic and prognostic information. Leveraging their pleiotropic actions, therapeutics targeting hypertension, heart failure, and metabolic syndrome have been developed [7,33–35].

For pre-clerkship education, ANP and BNP may be prioritised as the core teaching examples of cardiac endocrine function. Rather than requiring students to memorise BNP as an isolated laboratory value, early instruction can connect biomarkers to ventricular wall stress, cardiomyocyte secretion, renal-volume regulation, and dyspnoea evaluation. This prioritisation is important because natriuretic peptide physiology is complex, and early learners do not need exhaustive coverage of every peptide, receptor subtype, or downstream pathway during initial exposure.

### C-type natriuretic peptide (CNP)

CNP, a 22-amino acid peptide, is produced mainly by endothelial cells within the cardiovascular (CV) system, including cardiac endocardium. Unlike ANP and BNP, CNP primarily functions in a paracrine and autocrine manner rather than as a circulating hormone [6–8].

CNP plays a significant role in vascular homoeostasis by inducing vasodilation and inhibiting vascular smooth muscle proliferation [7]. Within the myocardium, it inhibits cardiac myocyte hypertrophy and cardiac fibroblast proliferation and collagen secretion. These actions contribute to the regulation of vascular tone and prevention of pathological remodelling, supporting vascular health [7]. CNP may be introduced in early medical education as a related natriuretic peptide that helps learners recognise that cardiac endocrine signalling exists on a spectrum from circulating endocrine effects to local paracrine and autocrine regulation.

### Receptor signalling and additional cardiac peptides

**Figure 1.**
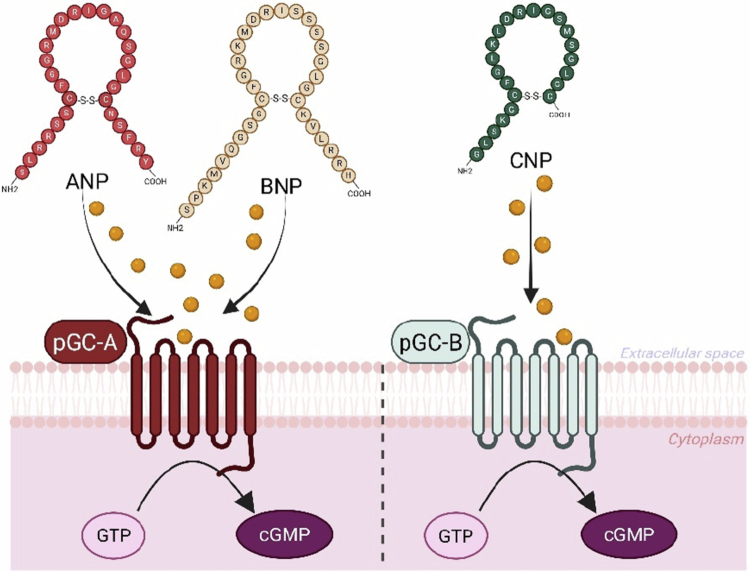
Receptor signalling pathways of cardiac natriuretic peptides. Atrial natriuretic peptide (ANP), B-type natriuretic peptide (BNP), and C-type natriuretic peptide (CNP) bind to particulate guanylyl cyclase receptors pGC-A and pGC-B, respectively, on target cell membranes. ANP and BNP activate pGC-A (also known as natriuretic peptide receptor-A, NPR-A), while CNP binds pGC-B (natriuretic peptide receptor-B, NPR-B). Ligand binding stimulates the intracellular guanylyl cyclase domain to convert guanosine triphosphate (GTP) to cyclic guanosine monophosphate (cGMP), the principal second messenger mediating natriuresis, vasodilation, and inhibition of pathological cardiac remodelling. The figure highlights the selective receptor interactions and downstream cGMP generation that underpin the endocrine regulation of cardiovascular and renal function by cardiac-derived peptides. Created in BioRender. Malsawmzuali, J. (2026) https:// BioRender.com/4yshrb3.

Natriuretic peptides exert their effects through natriuretic peptide receptors (NPRs). ANP and BNP primarily signal via NPR-A (guanylyl cyclase-A, GC-A), while CNP preferentially binds NPR-B (guanylyl cyclase-B, GC-B). Activation of these receptors increases intracellular cyclic guanosine monophosphate (cGMP) (Figure 1), which mediates vasodilation, renal sodium excretion, and anti-remodelling effects [8,33,36].

This guanylyl cyclase activity distinguishes NPR-A and NPR-B from the clearance receptor NPR-C, which primarily regulates peptide levels without generating cGMP [6,8,36]. Natriuretic peptides are also degraded by neprilysin, an enzymatic pathway that assumes clinical relevance when students encounter neprilysin inhibition in heart failure pharmacotherapy [23,24]. The selective activation of NPR-A/pGC-A by ANP and BNP and NPR-B/pGC-B by CNP underpins the heart’s hormonal regulation of CV and renal physiology [6,8,36].

Other hormones have been identified [37] as being produced by cardiac myocytes in varying degrees such as growth differentiation factor-15 (GDF-15) and myostatin, both members of the transforming growth factor-beta (TGF-*β*) cytokine superfamily [38]. These factors, produced in response to myocardial stress and injury, further support the concept of the heart as an active endocrine organ albeit with less central roles in educational frameworks [37,38].

Together, these peptides coordinate cardiovascular, renal, and metabolic homoeostasis through their systemic actions (Supplemental Figure 1).

## Cardiac endocrine function as a curricular opportunity in early medical education

Across the basic sciences, early medical education often presents content in discipline-specific or organ-system-based structures, and learners may experience these domains as fragmented when relationships across foundational mechanisms and clinical contexts are not made explicit [39–42]. This separation impairs students’ ability to transfer foundational knowledge to clinical reasoning, a phenomenon consistently documented across health professions education [43,44]. While each discipline contributes to essential concepts, their lack of explicit connection to one another and real-world patient scenarios can result in fragmented cognitive frameworks.

In anatomy, structural knowledge of the heart is typically emphasised in relation to its mechanical and electrophysiological roles, including chambers, valves, great vessels, coronary circulation, conduction pathways, and myocardial organisation [1,2,43]. These concepts are essential, but they can also serve as an entry point for teaching the heart’s endocrine function as a structure-function relationship. For example, atrial cardiomyocytes and their secretory granules provide a histologic basis for ANP synthesis and release, whereas ventricular wall stress provides an anatomical and physiological bridge to BNP secretion [6,8,21]. This observation is consistent with published analyses of anatomy curricula, which highlight reductions in laboratory time and shifts toward integrated instruction, but they provide limited visibility into session-level learning objectives and do not foreground cardiac endocrine function as a prominent anatomical concept [1,2]. Therefore, the available literature suggests that this topic is not consistently visible as an explicit structure-function concept in published curricular descriptions.

Similarly, work on physiology education documents the practical challenges of maintaining depth in core physiological mechanisms during curricular transitions [45]. Biochemistry covers molecular pathways in detail yet seldom ties them to specific cardiac structures or functional integration with other systems. As noted in broader curriculum reviews, mechanistic themes bridging molecular biology and organ-level physiology are frequently absent in pre-clerkship teaching [39,44]. This separation represents a missed opportunity to anchor biochemical mechanisms within anatomical structures and physiological processes relevant to patient care. Cardiac endocrine signalling is well suited to address this challenge because it connects atrial and ventricular structure, peptide hormone synthesis, receptor signalling, renal sodium handling, extracellular volume regulation, and clinical interpretation of BNP/NT-proBNP [6–8].

The consequences of this omission are well-documented in the broader literature on clinical reasoning and knowledge transfer [40,41]. Without deliberate integration, students struggle to develop robust mental models, such as illness scripts and conceptual schemas that link basic science to clinical decision-making [46–48]. This fragmentation diminishes the likelihood of successful ‘transfer out,’ where knowledge acquired in the classroom is applied to new clinical problems, and ‘transfer in,’ where prior knowledge facilitates learning new content. Studies of knowledge transfer in health professions education repeatedly demonstrate that insufficiently integrated curricula hinder students’ ability to reason mechanistically in clinical contexts [47–49]. As a result, learners may enter clerkships less prepared to connect patient presentations with underlying basic science mechanisms leading to diagnostic reasoning that is overly reliant on pattern recognition rather than mechanistic understanding.

The limited medical education literature directly addressing cardiac endocrine function further supports the need for a practical integration framework. The most directly applicable source emphasised that understanding the renal and cardiovascular effects of natriuretic peptides is important for first-year medical students, particularly because it helps learners interpret BNP levels in patients with heart failure [8]. This finding supports the educational relevance of this and also reinforces the need for clearer guidance on where, when, and how much natriuretic peptide physiology should be taught in pre-clerkship curricula.

Barriers contributing to this integration challenge are multifactorial. Curricular time pressures and competing instructional priorities may make it difficult to add clinically anchored, interdisciplinary content without displacing existing foundational material [39,45]. Faculty expertise misalignment, where basic science educators may lack direct clinical engagement and clinical faculty may not emphasise foundational mechanisms, limits opportunities for interdisciplinary reinforcement [39,45,50]. Additionally, there persists a perception that certain integrative topics are ‘too advanced’ for pre-clerkship students, despite evidence that early exposure supports both retention and clinical reasoning skill development [40,41,51,52]. For cardiac endocrine function, this concern can be addressed by prioritising a limited set of high-yield concepts rather than attempting exhaustive coverage. In early anatomy or histology, ANP can be introduced as an example of atrial structure-function integration. In cardiovascular physiology, BNP can be introduced as a clinically relevant marker of ventricular wall stress. More detailed discussion of CNP, natriuretic peptide receptor subtypes, cGMP signalling, neprilysin inhibition, and peptide-based therapeutics can be revisited later in physiology, biochemistry, pharmacology, or clinical cardiology teaching.

## Why early integration matters: educational and clinical perspectives

### Systems thinking starts here

Introducing the heart’s endocrine role early in the curriculum supports the development of systems thinking, the recognition that physiological systems are not isolated silos but interdependent networks. Teaching hormonal interactions between the cardiovascular, renal, and neuroendocrine systems at the outset fosters a foundational mindset of interconnectedness [39–41].

This approach is consistent with the lessons from Dundee’s integrated, systems-based spiral curriculum, which emphasised early exposure to system-level integration and repeated revisiting topics at increasing levels of complexity [53,54]. For cardiac endocrine function, a spiral approach allows students to first encounter the concept in anatomy or histology, revisit it in cardiovascular and renal physiology, and later apply it in clinical reasoning around volume overload, and heart failure. In contrast to traditional siloed learning, this method promoted sustained basic science knowledge across the curriculum and enabled students to build complexity on a strong foundation of interrelated concepts [46,53,54].

Moreover, integration in early phases, particularly horizontal integration across disciplines such as anatomy, physiology, and endocrinology, was viewed as essential to preparing students for clinical reasoning and problem-solving later in their education [39,55]. When taught in this way, the endocrine role of the heart becomes more than an isolated fact, it becomes a lens through which students view dynamic physiological interplay. An anatomy-centred sequence may be especially useful: students first learn the structural and histologic basis of cardiac endocrine function, then connect atrial stretch to ANP release and ventricular wall stress to BNP/NT-proBNP interpretation. This approach preserves primacy of cardiac anatomy whilst showing students that structure supports mechanical, electrical, and endocrine functions [6,8].

## Clinical relevance is immediate

BNP or NT-proBNP levels are commonly measured in the diagnosis and management of congestive heart failure [56,57]. Circulating BNP and NT-proBNP generally increase in response to myocardial wall stress, particularly ventricular pressure or volume overload, elevating their clinical extensions from mere isolated laboratory values [6,8,23]. Understanding its origin and function makes this biomarker meaningful, rather than memorising it. Introducing these peptides alongside early CV concepts potentially allows students to apply their knowledge clinically much sooner.

This clinical relevance can be introduced without overwhelming pre-clerkship learners. A concise case of a patient with dyspnoea, oedema, and elevated NT-proBNP can prompt students to ask why a ventricular hormone rises in heart failure, how wall stress produces a measurable blood marker, and why the kidney and vasculature respond to cardiac-derived peptides. Such a case links anatomy, physiology, renal sodium handling, and clinical decision-making in a single learning sequence. The direct educational value of this approach is supported by Wong et al., who emphasised that understanding the renal and cardiovascular effects of natriuretic peptides is important for first-year medical students because it helps them understand the significance of BNP levels in heart failure [8].

For early learners, BNP/NT-proBNP may be emphasised as a primary clinical application, while ANP can serve as the foundational structure-function example of atrial stretch and volume regulation. CNP, receptor subtypes, neprilysin inhibition, and peptide-based therapeutics can be introduced briefly or revisited in physiology, biochemistry, pharmacology, or clinical cardiology. This prioritisation addresses the practical concern that natriuretic peptide physiology is complex, and that pre-clerkship time is constrained.

### Enhances retention and engagement

Students are more likely to retain information and stay engaged when learning is framed within meaningful, real-world contexts [41,51,52]. Presenting the heart’s endocrine function in a patient-centred scenario such as a case study on acute decompensated heart failure, not only humanises the content but also aligns with principles of authentic learning, where relevance and application drive deeper understanding [22].

As Pawlina and Drake argue, authentic learning facilitates a shift from rote memorisation of anatomical or physiological facts to ‘dynamic tools for real-life problem-solving’ [22]. When students explore content that is immediately useful and tied to clinical relevance such as understanding how ANP informs heart failure management, they are more likely to engage with the material and apply it meaningfully in future clinical encounters​.

Authentic learning also supports ‘occupational realism,’ where students simulate the roles and reasoning patterns of practicing clinicians [50]. This includes working with real or simulated patient data, interpreting imaging or lab results, and discussing diagnoses within teams, thereby reinforcing both content mastery and professional identity formation. The integration of such scenarios early in the pre-clerkship curricula allows students to perceive knowledge not as isolated facts but as tools for patient care and interdisciplinary collaboration [22,50]. In this context, the endocrine function of the heart offers a compact, high-yield example: students can move from atrial and ventricular structure to peptide secretion, to renal and vascular effects, to interpretation of a biomarker used in clinical practice.

### Practical strategies for curriculum integration

To effectively teach the heart’s endocrine function, content can be integrated across multiple basic science disciplines. Rather than adding a separate curricular block, we recommend an anatomy-anchored, spiral approach in which students first encounter the endocrine function of the heart through cardiac structure and histology, then revisit the concept in cardiovascular physiology, biochemistry, pharmacology, and clinical reasoning [22,39,53,54]. Table 1 outlines practical strategies for incorporating natriuretic peptide biology into anatomy, physiology, and biochemistry courses, emphasising clinical relevance and interdisciplinary learning. The recommendations in Table 1 are author-derived and informed by the literature reviewed, rather than direct recommendations from any single included study.

However, implementation may face challenges including curriculum overcrowding and faculty expertise misalignment [39,45,54]. Interdisciplinary sessions, clinician involvement, and concise, case-driven teaching modules can help overcome these barriers while enhancing student engagement and knowledge transfer [42,51,52]. In anatomy and histology, a brief structure-function teaching point can introduce atrial cardiomyocyte secretory granules and chamber-specific peptide release. In cardiovascular physiology, this foundation can be expanded to include stretch-mediated ANP/BNP release and renal-volume regulation. In clinical sessions, students can apply the concept to dyspnoea, congestion, ventricular wall stress, and BNP/NT-proBNP interpretation. This staged approach aligns with integrated curriculum design principles, authentic learning, and early clinical exposure models described in medical education [22,42,51–54].

**Table 1.** Practical strategies for curriculum integration across basic sciences.

Discipline	Key Concepts and recommended depth	Suggested Integration Strategies	Clinical/Translational Link
Anatomy and histology	Introduced as anatomical entry point, ANP is initial structure-function example. Atrial and ventricular myocyte histology. Secretory granules, juxtanuclear zones. Structural adaptations for peptide secretion.	Microscopy lab with annotated slides. 3D models highlighting atrial/ventricular differences. Case-based discussion linking structure to function. Brief ‘heart as endocrine organ’ teaching point during cardiac anatomy.	Connect atrial stretch to ANP release; introduce ventricular wall stress as the structural basis for BNP/NT-proBNP elevation in HF.
Cardiovascular Physiology	Natriuretic peptide signalling (ANP, BNP, CNP). Hormonal regulation of blood pressure, fluid, and electrolyte balance. Receptor mechanisms (NPR-A/B, cGMP)	Concept maps linking cardiac stretch to renal and vascular responses; short clinical vignette on volume overload; interactive diagrams of heart-kidney signalling; integration with blood pressure and extracellular volume regulation.	Explains why the heart participates in renal and vascular regulation; prepares students to understand BNP/NT-proBNP testing in HF. Application in diagnosing/monitoring HF. Explains physiological rationale for diuretics and vasodilators.
Renal and endocrine physiology	Reinforce natriuretic peptides as counter-regulatory hormones opposing sodium and volume retention; integrate with RAAS, aldosterone, vasopressin, GFR, and sodium handling.	Compare RAAS and natriuretic peptide effects in a two-column exercise, case discussion on oedema or volume overload.	Helps students understand volume homoeostasis, oedema, congestion, and the endocrine crosstalk between heart, kidney, vasculature, and neuroendocrine systems.
Biochemistry and pharmacology	Peptide hormone biosynthesis and processing; NPR-A/NPR-B receptor signalling; cGMP as a second messenger; neprilysin degradation. Post-translational modification cGMP-dependent signalling cascades	Flowcharts tracing synthesis → secretion → receptor binding Problem-based learning exercises linking molecular signalling to therapy. Drug-mechanism exercises on neprilysin inhibition.	Links molecular pathways to therapeutic targets (e.g., MANP, sacubitril/valsartan). Reinforces mechanistic understanding of clinical biomarkers.
Clinical Integration	Natriuretic peptides as biomarkers. Interpretation of BNP/NT-proBNP in HF, PH, valvular disease. Implications for drug selection and patient monitoring.	Bedside simulation or case-based discussion. Interdisciplinary teaching with cardiologists.	Directly applies basic science knowledge to clinical decision-making. Demonstrates translational relevance of endocrine function.

This table summarises practical approaches to incorporating the endocrine role of the heart across major basic science disciplines. The proposed sequence prioritises ANP and BNP/NT-proBNP for early learners because these peptides directly connect cardiac structure, myocardial stretch, renal-volume regulation, and heart failure evaluation. ANP = Atrial Natriuretic Peptide; BNP = B-type Natriuretic Peptide; CNP = C-type Natriuretic Peptide; NPR = Natriuretic Peptide Receptor; GC = Guanylyl Cyclase; cGMP = cyclic Guanosine Monophosphate; HF = Heart Failure; PH = Pulmonary Hypertension; RAAS = Renin–Angiotensin–Aldosterone System.

### Cardiologist perspective: bridging basic science with clinical decision-making

The heart’s endocrine signals are critical bedside guides. In a 45-year-old with ambiguous dyspnoea, an NT-proBNP of 3,500 pg/mL provides strong evidence of ventricular wall stress and supports the diagnosis of heart failure when interpreted in clinical context, especially when the physical exam is limited by habitus [6,8,23,57]. This molecular insight triggers rapid diuresis and reduces hospital stays. This foundational physiology drove the landmark PARADIGM-HF trial, where sacubitril/valsartan decreased CV death and HF hospitalisations by 20% [23,24,58]. Sacubitril/valsartan is an angiotensin receptor-neprilysin inhibitor (ARNI) that simultaneously augments beneficial natriuretic peptide signalling, by inhibiting neprilysin-mediated degradation of natriuretic peptides, and suppresses the renin-angiotensin-aldosterone system (RAAS) through angiotensin II receptor blockade [24,57].

This endocrine role also fuels ‘designer’ therapeutics. MANP, a subcutaneous ANP analogue, increases cGMP, lowers blood pressure, and suppresses aldosterone in humans [33]. CRRL 094, a bispecific peptide, enhances natriuretic and antifibrotic effects in preclinical models [58–60]. These breakthroughs demonstrate how understanding the cardiac endocrine role translates directly into cutting-edge CV care.

Beyond heart failure, natriuretic peptides have expanded roles. In ischaemic heart disease, they predict remodelling and mortality [61,62]. In valvular disease, they help guide timing of intervention, especially in asymptomatic cases [62–65]. In pulmonary hypertension, BNP/NT-proBNP levels track pressure, RV function, and prognosis across adults and children, even in congenital heart disease and scleroderma [65–70]. Even in oncology, BNP acts as a sentinel for early chemotherapy-induced cardiotoxicity, helping shape clinical care [71].

By pairing molecular signalling with cardiac anatomy, medical education reframes the heart as an integrative hemodynamic and endocrine organ that directs systemic physiology and informs precision therapy.

## Discussion

The findings of this narrative review highlight an educational opportunity at the interface of contemporary cardiovascular science and early medical education. Although the endocrine function of the heart is now well established within cardiovascular physiology and clinical practice [3,4,6,7], it is not consistently foregrounded as an explicit structure-function concept in published discussions of foundational medical curricula. The available literature does not allow us to conclude that cardiac endocrine function is absent from medical school curricula; rather, it suggests limited explicit guidance on how this content should be introduced, sequenced, and reinforced for pre-clerkship learners. This limited explicit framing may reduce opportunities for learners to integrate structure, physiology, and molecular mechanisms in ways that support mechanistic clinical thinking.

The literature review suggests that early exposure to integrative physiological concepts may strengthen conceptual frameworks that learners rely on during clinical training [40,41]. When foundational science is presented in isolation, students may struggle to connect to molecular mechanisms with organ-level function and clinical manifestations [46,48,49]. In contrast, curricular approaches that use clinically relevant examples, such as BNP/NT-proBNP interpretation in heart failure, to link cardiac structure, peptide secretion, renal-volume regulation, and bedside decision-making, appear to support systems thinking and knowledge transfer across contexts [8,23,41]. These observations align with established educational frameworks emphasising cognitive integration, spiral curriculum design, and authentic learning [22,40,53,54].

From an educational perspective, the endocrine role of the heart represents a particularly suitable model for interdisciplinary integration. The natriuretic peptide system spans anatomy, histology, physiology, biochemistry, and clinical medicine, allowing educators to illustrate how molecular signalling emerges from specific cardiac structures and informs real-world decision making [6–8,21]. This review adds to the field by proposing an anatomy-anchored framework for teaching cardiac endocrine function: students first encounter the structural and histologic basis of hormone secretion, then revisit ANP and BNP/NT-proBNP in cardiovascular and renal physiology, and later apply these concepts to heart failure diagnosis, pharmacology, and clinical reasoning. Integrating such content early in the curriculum may help learners view foundational knowledge as a functional tool rather than a collection of facts.

At the same time, the literature also identifies practical challenges to curricular integration, including limited curricular time, competing instructional priorities, and variability in faculty expertise [39,45,54]. Studies describing successful integration efforts emphasise the importance of focused, clinically anchored teaching modules and collaboration between basic science and clinical faculty [42]. Rather than requiring wholesale curricular redesign, these approaches suggest that targeted integration at key instructional points may be both feasible and educationally meaningful. For cardiac endocrine function, the most practical approach may be selective prioritisation: ANP can be used as the initial anatomy/histology example of atrial stretch and endocrine secretion, whereas BNP/NT-proBNP can serve as the primary clinical example linking ventricular wall stress to heart failure evaluation. More detailed treatment of CNP, receptor subtypes, cGMP signalling, neprilysin inhibition, and designer natriuretic peptides can be revisited later as learners develop more advanced physiological and pharmacological frameworks.

Importantly, the conclusions drawn in this review reflect synthesis of existing literature rather than empirical evaluation of a specific curricular intervention. Because this was a narrative review, it was not designed to quantify curricular coverage across institutions, map all medical school learning objectives, or determine whether cardiac endocrine function is taught in individual courses. Publicly available curricular descriptions may not capture session-level objectives, lecture content, small-group cases, or assessment items. Further research examining learner outcomes, retention, and clinical reasoning following early integration of cardiac endocrine physiology would help clarify its educational impact. Such work could also inform best practices for integrating complex physiological concepts into pre-clerkship curricula without increasing cognitive workload. Future studies could evaluate brief anatomy or histology-based teaching interventions, interdisciplinary cardiovascular-renal-endocrine cases, or assessment items that require students to connect cardiac structure with natriuretic peptide physiology and clinical biomarker interpretation.

## Conclusion

The heart’s endocrine function is not just a fascinating scientific discovery; it is a cornerstone of modern cardiology with profound clinical and educational implications [3,4,6,7,56]. Yet, published medical education literature provides limited explicit guidance on how this aspect of cardiac biology should be incorporated in early medical education, particularly within anatomy-centred and pre-clerkship curricula [8]. Integrating this knowledge into the pre-clerkship curricula can help to cultivate a truly holistic understanding of cardiovascular physiology by linking cardiac anatomy with myocardial stretch, natriuretic peptide signalling, renal-volume regulation, and clinical biomarker interpretation [6,8]. Early exposure to the heart’s hormonal role may help students to connect foundational science with real-world clinical applications, enhancing critical thinking and systems approach from the very start of their training [22,40,41]. By embracing targeted curriculum integration that includes the heart’s endocrine functions, medical education can better equip future clinicians to meet the complex demands of CV health, ultimately improving patient outcomes and advancing healthcare. The heart’s endocrine story therefore offers not a separate topic to add, but an anatomy-anchored model for integrating foundational science with clinical medicine.

## Supplementary Material

Supplemental_Figure.docx Supplemental_Figure.docx

## Data Availability

Not applicable.
