# Supplementary material for: Beyond the pump: integrating the heart’s endocrine function into early medical education
Source: Med Educ Online. 2026 Jul 15;31(1):2704285. doi: 10.1080/10872981.2026.2704285 (PMC13374749; doi:10.1080/10872981.2026.2704285)
Supplement: Supplemental_Figure.docx [file ZMEO_A_2704285_SM8132.docx]

**Supplemental Figure:**

**
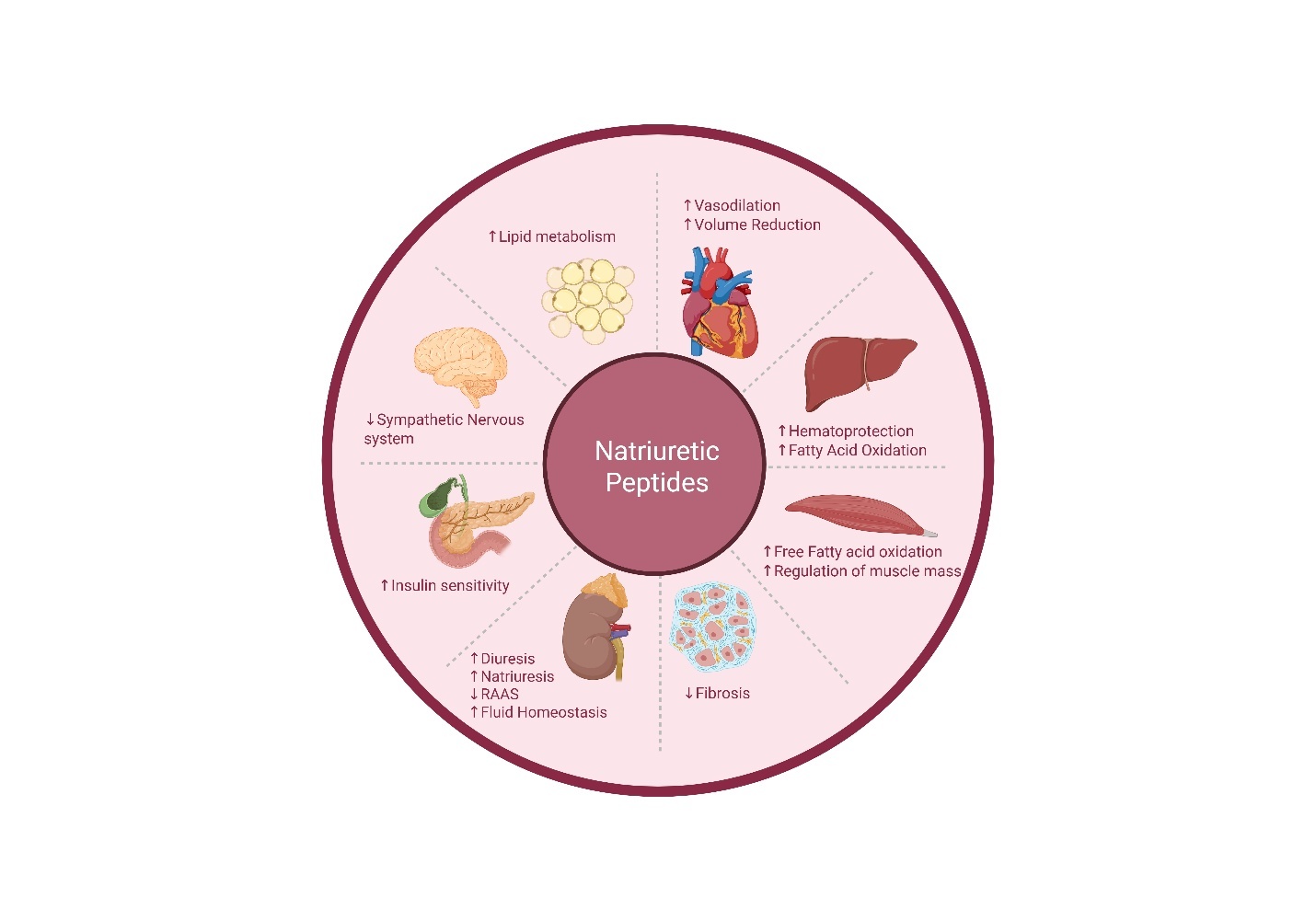
**

**Supplemental Figure 1. Systemic physiological actions of natriuretic peptides**

Overview of the multisystem effects of cardiac-derived natriuretic peptides across major organ systems. Natriuretic peptides enhance glomerular filtration rate (GFR), natriuresis, diuresis, and fluid homeostasis while inhibiting the renin–angiotensin–aldosterone system (RAAS) in the kidney. In the heart and vasculature, they promote vasodilation, volume reduction, and protection against hypertrophy and remodeling. In adipose tissue, they stimulate lipid metabolism; in skeletal muscle, they increase fatty acid oxidation and regulate muscle mass; and in the liver, they support hematoprotection and fatty acid utilization. Additional systemic effects include reduced sympathetic tone and increased insulin sensitivity. Collectively, these mechanisms illustrate the endocrine role of the heart in coordinating cardiovascular, renal, and metabolic homeostasis.

" Created in BioRender. Malsawmzuali, J. (2026) <https://BioRender.com/4yshrb3>".
